# Abnormalities in normal-appearing white matter from which multiple sclerosis lesions arise

**DOI:** 10.1093/braincomms/fcab176

**Published:** 2021-08-10

**Authors:** Colm Elliott, Parya Momayyezsiahkal, Douglas L Arnold, Dawei Liu, Jun Ke, Li Zhu, Bing Zhu, Ilena C George, Daniel P Bradley, Elizabeth Fisher, Ellen Cahir-McFarland, Peter K Stys, Jeroen J G Geurts, Nathalie Franchimont, Arie Gafson, Shibeshih Belachew

**Affiliations:** 1 NeuroRx Research, Montreal, QC H2X 3P9, Canada; 2 McGill University, Montreal, QC H3A 0G4, Canada; 3 Biogen Digital Health, Biogen, Cambridge, MA 02142, USA; 4 Biogen, Cambridge, MA 02142, USA; 5 Department of Neurology, Massachusetts General Hospital, Boston, MA 02114, USA; 6 Department of Clinical Neurosciences, Hotchkiss Brain Institute, Cumming School of Medicine, University of Calgary, Calgary, AB T2N 4N1, Canada; 7 Department of Anatomy and Neurosciences, Amsterdam UMC, 1081 HV Amsterdam, Netherlands

**Keywords:** relapsing multiple sclerosis, progressive multiple sclerosis, MRI, white matter lesions, demyelination

## Abstract

Normal-appearing white matter is far from normal in multiple sclerosis; little is known about the precise pathology or spatial pattern of this alteration and its relation to subsequent lesion formation. This study was undertaken to evaluate normal-appearing white matter abnormalities in brain areas where multiple sclerosis lesions subsequently form, and to investigate the spatial distribution of normal-appearing white matter abnormalities in persons with multiple sclerosis. Brain MRIs of pre-lesion normal-appearing white matter were analysed in participants with new T2 lesions, pooled from three clinical trials: SYNERGY (NCT01864148; *n* = 85 with relapsing multiple sclerosis) was the test data set; ASCEND (NCT01416181; *n* = 154 with secondary progressive multiple sclerosis) and ADVANCE (NCT00906399; *n* = 261 with relapsing-remitting multiple sclerosis) were used as validation data sets. Focal normal-appearing white matter tissue state was analysed prior to lesion formation in areas where new T2 lesions later formed (pre-lesion normal-appearing white matter) using normalized magnetization transfer ratio and T2-weighted (nT2) intensities, and compared with overall normal-appearing white matter and spatially matched contralateral normal-appearing white matter. Each outcome was analysed using linear mixed-effects models. Follow-up time (as a categorical variable), patient-level characteristics (including treatment group) and other baseline variables were treated as fixed effects. In SYNERGY, nT2 intensity was significantly higher, and normalized magnetization transfer ratio was lower in pre-lesion normal-appearing white matter versus overall and contralateral normal-appearing white matter at all time points up to 24 weeks before new T2 lesion onset. In ASCEND and ADVANCE (for which normalized magnetization transfer ratio was not available), nT2 intensity in pre-lesion normal-appearing white matter was significantly higher compared to both overall and contralateral normal-appearing white matter at all pre-lesion time points extending up to 2 years prior to lesion formation. In all trials, nT2 intensity in the contralateral normal-appearing white matter was also significantly higher at all pre-lesion time points compared to overall normal-appearing white matter. Brain atlases of normal-appearing white matter abnormalities were generated using measures of voxel-wise differences in normalized magnetization transfer ratio of normal-appearing white matter in persons with multiple sclerosis compared to scanner-matched healthy controls. We observed that overall spatial distribution of normal-appearing white matter abnormalities in persons with multiple sclerosis largely recapitulated the anatomical distribution of probabilities of T2 hyperintense lesions. Overall, these findings suggest that intrinsic spatial properties and/or longstanding precursory abnormalities of normal-appearing white matter tissue may contribute to the risk of autoimmune acute demyelination in multiple sclerosis.

## Teaser

Elliott et al. report that the spatial distribution of normal-appearing white matter (NAWM) abnormalities in people living with multiple sclerosis (MS) largely recapitulates the anatomical mapping of probabilities of MS lesions while longstanding precursory abnormalities of NAWM tissue are observed in specific regions where MS lesions will ultimately form.

## Graphical abstract

**Graphical Abstract:**
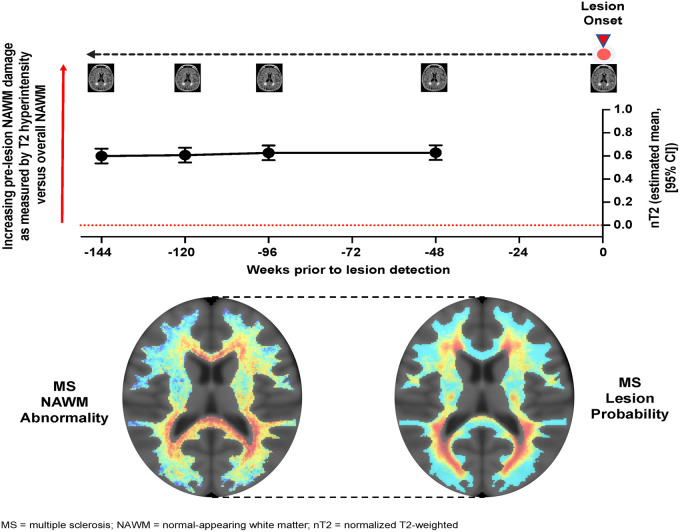

## Introduction

MRI is an established tool for diagnosing multiple sclerosis and monitoring disease activity and is a validated outcome measure used in clinical trials for multiple sclerosis.^1–3^ Advances in MRI technology have provided biomarkers that are indicative of both underlying pathologic processes and the temporal dynamics of disease progression.^4^ These advances include novel demonstrations of multiple sclerosis pathology, such as leptomeningeal enhancement^5–7^ and slowly expanding lesions,^8^ myelin quantification,^9^^,^^10^ and microstructural tissue damage.^11^

MRI has also shown that ‘normal-appearing’ white matter (NAWM) is diffusely abnormal in the CNS of patients with multiple sclerosis, and that these abnormalities are not distributed uniformly throughout NAWM.^12^^,^^13^ However, the spatial mapping of NAWM abnormalities in multiple sclerosis and their relationship to existing lesions and risk of subsequent lesion formation remains to be fully elucidated.^14^^,^^15^ *In vivo* MRI abnormalities have been identified prior to the appearance of new inflammatory lesions. These include reduced magnetization transfer ratio (MTR),^16–19^ increased mean diffusivity,^20^ increased average apparent diffusion coefficient,^21^^,^^22^ increased choline peaks,^23^ altered magnetic susceptibility^24^ and pre-enhancement signal abnormalities.^25^ Most of these abnormalities were observed in the weeks and months prior to lesion formation, although some were observed as far back as 18 months in advance.^18^ Limitations of these previous studies include their small sample sizes, short retrospective follow-up duration and lack of multivariate analysis.

The formation of new lesions is a pathological hallmark of disease activity in multiple sclerosis.^26^ The precise pathology underlying pre-lesion abnormalities and their spatial distribution in the brain NAWM have not been characterized, although it is hypothesized that they may represent primary demyelination or altered properties of axons and/or myelin in the absence of macroscopic structural damage, perivascular inflammation or astrocytic proliferation.^27^ Forward and backward analyses of new lesion formation on MRI may elucidate processes involved in the evolution of individual lesions, including the assessment of abnormalities in CNS tissue state prior to the earliest stages of detectable lesion formation.^26^

In this study, brain MRI was used to assess the spatial pattern of NAWM abnormalities versus probabilities of lesions in PwMS. To clarify the spatial and temporal relationship between prior NAWM abnormalities and subsequent lesion formation, the specific NAWM areas from which lesions arise was analysed in three separate clinical trial populations encompassing a total of 1280 new T2 lesions from 500 patients with relapsing-remitting multiple sclerosis (RRMS) and secondary progressive multiple sclerosis (SPMS), with up to 144 antecedent weeks of longitudinal pre-lesion data.

## Materials and methods

### Trial design and MRI procedures

**Figure 1:**
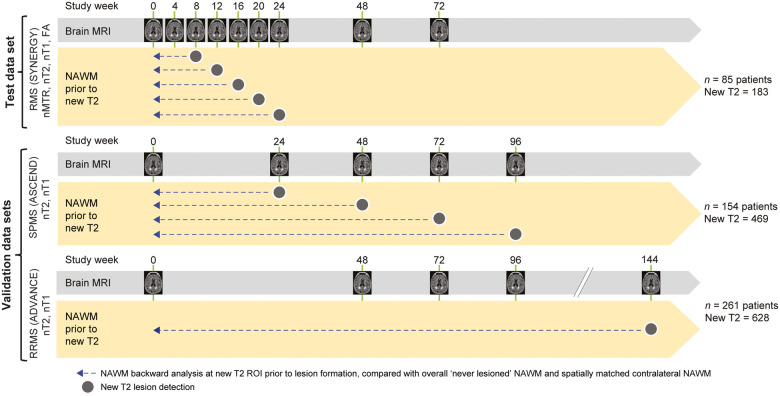
Backward analysis of NAWM integrity prior to new T2 lesion formation. FA, fractional anisotropy.

An analysis of MRI changes in NAWM preceding new T2 lesion formation was performed using patients with evaluable new T2 lesions from three clinical trial populations (Fig. 1). The pooled population (placebo and treatment groups) of the SYNERGY study (NCT01864148; *n* = 85 patients with 183 new T2 lesions) was used as a test data set of patients with relapsing multiple sclerosis (RMS), while the larger studies, ASCEND (NCT01416181; *n* = 154 patients with SPMS with 469 new T2 lesions) and ADVANCE (NCT00906399; *n* = 261 patients with RRMS with 628 new T2 lesions) were used as validation data sets. In the SYNERGY test data set, new T2 lesions were detected at Weeks 8, 12, 16, 20 and 24, and pre-lesion NAWM was analysed on monthly scans up to 24 weeks prior to lesion formation. In the validation data sets, new T2 lesions were detected at Weeks 24, 48, 72 and 96 in the ASCEND trial and at Week 144 in the ADVANCE trial, and pre-lesion NAWM was analysed on semi-annual or annual scans for up to 2 years prior to lesion formation.

The actual time of lesion onset for all new T2 lesions could not be known exactly and may have occurred anywhere between the scan where new T2 lesions were first observed and the prior reference scan. In SYNERGY, where the scans were acquired every 4 weeks during the analysis period, the actual lesion onset will have occurred anywhere from 0 to 4 weeks prior to first observation of the new lesion. The time point immediately prior to lesion observation (–4 weeks) was not included in the analysis to exclude any assessment of new T2 lesions potentially very close to actual lesion onset. In ADVANCE and ASCEND, the offset between observed and actual lesion onset is larger due to longer scanning intervals and will be anywhere from 0 to 24 weeks in ASCEND and 0 to 48 weeks in ADVANCE.

SYNERGY was a double-blind, placebo-controlled, dose-ranging, parallel-group, phase II study where patients with RMS were randomly allocated in a 1:2:2:2:2 ratio to one of five parallel treatment groups of opicinumab (3, 10, 30 or 100 mg/kg) or placebo once every 4 weeks, with added-on intramuscular interferon beta-1a as background anti-inflammatory treatment once a week, over 72 weeks.^28^ ASCEND was a double-blind, placebo-controlled, parallel-group, phase III study where patients with SPMS were randomly assigned (1:1) to receive 300 mg intravenous natalizumab or placebo every 4 weeks for 2 years.^29^ ADVANCE was a double-blind, placebo-controlled, parallel-group, 2-year, phase III study with open-label extension where patients with RRMS were randomized (1:1:1) to placebo or 125 µg peginterferon beta-1a every 2 or 4 weeks.^30^ A pooled-treatment group approach was selected for this study given the overall low sample size of the placebo groups across all trials while accounting for treatment effects in the multivariate model analysis.

Details for the brain MRI acquisitions for each trial data set are described in Supplementary Material-1.

### Backward analysis of NAWM integrity prior to new lesion formation

New T2 lesions were automatically identified at pre-selected time points,^31^ with respect to the previous time point, and tracked longitudinally from lesion onset until end of study.^32^ All automatically identified new T2 lesions were manually reviewed and any falsely detected lesions were removed. Areas corresponding to unresolved portions of new T2 lesions remaining at end of study (residual new T2) were used as initial definitions of pre-lesion regions of interest (ROIs) (Supplementary Figure). Cross-sectional segmentation of T2 lesions was performed on all scans using a semi-automated approach, where an initial automatic segmentation of T2 hyperintense lesions was manually corrected by trained readers. Automatic detection of T2 hyperintense lesions was performed using a Bayesian classifier providing probabilistic tissue classification at each voxel based on multi-sequence MRI intensities and atlas-derived prior probabilities of healthy tissue.^33^^,^^34^ NAWM masks were generated using a similar Bayesian approach. The manually reviewed T2 lesion masks were used as input to ensure all T2 lesions were excluded from the resultant NAWM mask.^34^

To reduce the influence of surrounding lesions and/or previous focal demyelination in the same area, only pre-lesion ROIs that met the following criteria were considered for further analysis: (i) at least 10 voxels (30 mm^3^) in size; (ii) classified as NAWM at all time points prior to lesion onset; and (iii) at least 2 mm away from any pre-existing lesion at all time points prior to lesion onset.

For each pre-lesion ROI, the corresponding contralateral NAWM ROI was determined by reflecting the pre-lesion ROI across the midline of the brain. Contralateral ROIs were adjusted to ensure that only portions that were classified as NAWM and at least 2 mm from T2 lesions at all time points were considered as spatially matched contralateral control tissue. Any adjustments made to contralateral ROIs were also applied to the pre-lesional ROI via reflection across the midline. Matched contralateral and pre-lesion ROI pairs of at least 10 voxels (30 mm^3^) in size were retained for subsequent analysis. The process for determining pre-lesion and contralateral ROIs is shown graphically in Supplementary Figure.

**Figure 2:**
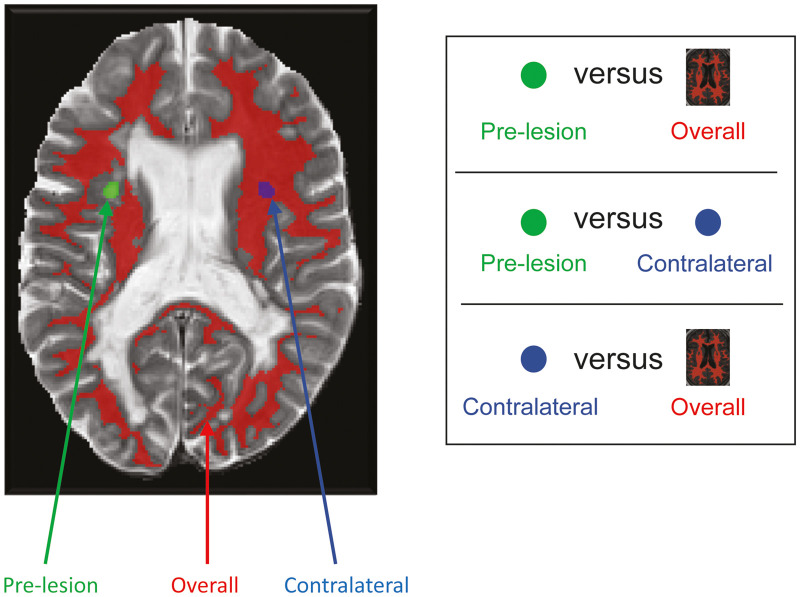
Types of pairwise differences in measures of NAWM tissue integrity prior to new T2 lesion formation. Based on nT2 and nT1 intensity measures of NAWM tissue integrity.

Overall, NAWM corresponds to tissue classified as NAWM at all time points and at least 2 mm from any voxel classified as T2 lesion at any study time point. Three types of pairwise differences in normalized MTR (nMTR), normalized T2-weighted (nT2) and normalized T1-weighted (nT1) intensity were defined as outcomes of interest to study NAWM tissue integrity: pre-lesion versus overall; pre-lesion versus contralateral; and contralateral versus overall (Fig. 2). Because nMTR in NAWM varies spatially throughout the brain (but symmetrically across the midline), the pre-lesion versus contralateral comparison is used to control for this spatial variability and quantify pre-lesion abnormalities that are associated with a specific new T2 lesion event.

### Spatial analysis of NAWM abnormalities and probabilities of lesion location

Two brain atlases of nMTR in NAWM were constructed, representing voxel-wise mean nMTR in NAWM in persons with multiple sclerosis (PwMS; SYNERGY, *n* = 419) and healthy controls (HCs; SYNERGY scanner-matched dummy runs, *n* = 110), respectively. Atlases were constructed by performing non-linear registration of each patient to International Consortium for Brain Mapping (ICBM) space and resampling the corresponding subject masks for NAWM and baseline nMTR images into ICBM space. Each atlas of nMTR in NAWM was constructed by averaging resampled nMTR within the resampled NAWM masks over all patients in the respective cohorts (PwMS or HCs). A NAWM abnormality atlas was then generated by subtracting the nMTR in NAWM atlases for HCs and PwMS, providing a voxel-wise representation of mean nMTR differences in NAWM between HCs and PwMS.

An overall brain T2 lesion atlas was constructed, representing the probability of T2 hyperintense lesion occurrence at each voxel in the brain. The atlas was generated by non-linearly registering baseline total T2 hyperintense lesion masks (SYNERGY, *n* = 419) to ICBM space, summing the resampled T2 lesion masks and dividing by the total number of patients. A representation of the overall brain T2 lesion atlas discretized in four categorical bins of increasing probabilities was also generated to facilitate comparisons between atlases of NAWM abnormalities and T2 lesion probability.

### Normalization of MTR, T2-weighted and T1-weighted signal intensity measures

MTR intensities were calibrated using a previously described method,^35^ where median MTR values for both grey and white matter were determined for a reference HC for each scanner and subsequently used as scanner-specific normalization factors for all subsequent patient scans on the same scanner (dummy-run participants, total number of scanners = 110). For each new patient scan acquired on the same scanner, the MTR value corresponding to the median HC grey matter was mapped to 0, while the MTR value corresponding to the median HC white matter was mapped to 1. In normalized MTR images, a value of 0 can thus be interpreted as corresponding to healthy (i.e. without multiple sclerosis) grey matter, while a value of 1 can be interpreted as corresponding to healthy white matter.

To assess nT1 intensity change over time, T1-weighted intensities were normalized in a two-stage process: (i) a tissue-based normalization was performed in baseline T1-weighted scans, where intensities for a given patient were linearly normalized by mapping the median grey matter T1 intensity to a value of 0, and mapping the median NAWM intensity to 1 and (ii) a longitudinal normalization was performed for all subsequent T1-weighted scans, where intensities of a given patient were normalized to the baseline T1-weighted scan using least-trimmed squares, which performs linear regression between co-registered sequential scans using the 50% of voxels whose least-squares fit possesses the smallest sum of squared residuals.^36^ This normalizes intensities over time within a given patient based only on the subset of voxels that remain relatively unchanged over time. The first stage performs a cross-sectional normalization providing a comparable scale for measures of T1 intensity change across different patients, while the second stage provides a longitudinal normalization that minimizes acquisition-related intensity variation across time for a given patient.

To assess nT2 intensity change over time, T2-weighted intensities were also normalized in a two-stage process, with (i) least-trimmed squares used to normalize follow-up images to the baseline image, and (ii) mean-normalization to NAWM, such that mean NAWM is mapped to a value of 0 and 1 unit of normalized T2-weighted intensity represents one intensity standard deviation of NAWM.^37^ A tissue-based normalization using both NAWM and grey matter was not used for T2-weighted images due to reduced contrast between NAWM and normal-appearing grey matter on T2-weighted images as compared to T1-weighted images.

### Statistical analysis

For the main analysis of NAWM tissue integrity prior to new T2 lesion formation, the outcomes were three pairwise differences in mean intensities (Fig. 2) at each available time interval prior to new lesion formation. SYNERGY was used as the test data set, and ASCEND and ADVANCE were used as validation data sets. Each longitudinal outcome was analysed using linear mixed-effects models. Time from lesion onset, which was analysed as a categorical variable, patient-level characteristics (including treatment group) and other pre-treatment baseline variables were treated as fixed-effects. Lesion and patient were treated as random effects, and the random lesion effect was nested under random patient effect. First-order autoregressive variance–covariance matrix AR(1) was used to model the potential correlation of outcome measures over time. Such a covariance structure assumes that correlations between measurements are the highest for adjacent times, and it assumes a systematically decreasing correlation with increasing distance between time points. To account for the impact of unequal number of lesions from different patients, a weight was created that equalled the inverse of the number of lesions for each patient and was used in the linear mixed-effects analysis. Type I error rate of 0.05 was assumed for all hypothesis testing and evaluation of significance of variables. Multiple comparison adjustment was made when hypothesis testing was conducted on multiple levels of a categorical variable.

For the evaluation of the relationship between categorical bins of spatial probabilities of T2 hyperintense lesions and NAWM abnormalities as measured by nMTR difference (HC-PwMS), as an approximately linear trend was observed over the bins of increasing probabilities, a linear trend test was performed on the nMTR difference over the probability bins. All statistical analyses were conducted using the software package R (v3.6.2). Linear mixed-effect analysis was conducted with package ‘nlme’ (v3.1), least-square means were calculated using package ‘lsmeans’ (v2.30) and effect size was calculated using package ‘effect size’ (v0.4) (https://cran.r-project.org/web/packages/nlme/index.html. Accessed August 26, 2021).

### Data availability

Requests for data should be submitted via the Biogen Clinical Data Request Portal (www.biogenclinicaldatarequest.com). To gain access, data requestors will need to sign a data-sharing agreement. Data are made available for 1 year on a secure platform.

## Results

### Demographic and disease characteristics of patients with multiple sclerosis

Differences in patient characteristics across trials were observed (Supplementary Table), as expected due to different enrolment criteria for each trial. Patients with RMS from SYNERGY and patients with RRMS from ADVANCE were younger than the SPMS population of ASCEND. The sex ratio was comparable across all three trial data sets. The baseline level of acute MRI inflammation as measured by T1 gadolinium-enhancing (Gd^+^) lesions was greater in the RMS and RRMS populations (SYNERGY and ADVANCE, respectively) than in patients with SPMS (ASCEND). Conversely, the total T2 lesion volume burden was nearly 2-fold higher in the SPMS population compared to the RMS and RRMS populations (Supplementary Table).

### Analysis of NAWM tissue integrity prior to new T2 lesion formation in the test data set

**Figure 3:**
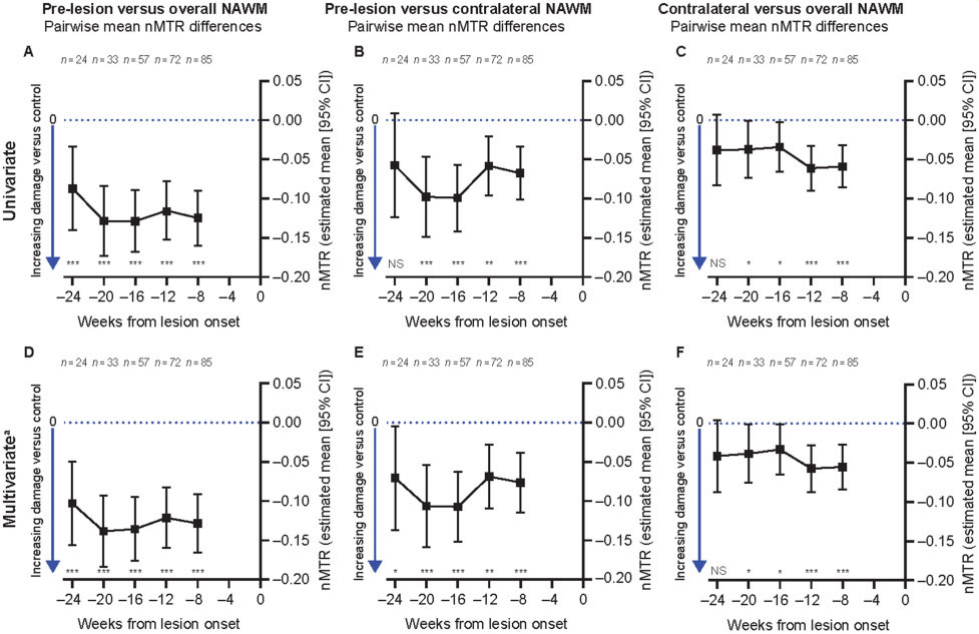
**Weighted mixed-effects model analysis of pre-lesion NAWM nMTR before new T2 lesion onset in RMS (test data set).** Lower nMTR in pre-lesion NAWM as compared to overall NAWM (**A, D**) and as compared to spatially matched contralateral NAWM (**B, E**). Significantly lower nMTR was also seen at all time points from Week 8 to Week 20 in spatially matched contralateral NAWM as compared to overall NAWM (**C, F**). NS, not significant. ^a^Model adjusted for age, sex, age:sex interaction, treatment group (placebo, opicinumab 100 mg/kg, 30 mg/kg, 10 mg/kg, 3 mg/kg), multiple sclerosis disease duration, time since new T2 lesion onset, residual volume of new T2 lesion, baseline T1 Gd^+^ lesion count and baseline non-enhancing total T2 hyperintense lesion volume. The multivariate model could not account for potential courses of steroid administration although this variable is unlikely to be at play in this analysis as, per protocol, if a subject’s study MRI was scheduled to occur <4 weeks after the last dose of a steroid treatment for a relapse, the MRI was rescheduled, so that it was performed either before the steroid treatment or between 4 and 8 weeks after the last dose of steroid treatment. *P < 0.05, **P < 0.01, ***P < 0.001. All tests were based on the least-square means estimates from the weighted mixed-effects models. For complete model output, see Supplementary material-2.

Based on a univariate patient-weighted mixed-effects model analysis, a lower nMTR in pre-lesion NAWM was observed as compared to overall NAWM at all time points from Week 8 to Week 24 prior to new T2 lesion onset in patients with RMS from the SYNERGY trial test data set (Fig. 3A). A significantly lower nMTR was also observed in pre-lesion NAWM compared to spatially matched contralateral NAWM at all time points from Week ‒8 to Week ‒20 prior to new T2 lesion onset (Fig. 3B), whereas differences were non-significant at Week ‒24.

In a multivariate mixed-effects model analysis (adjusted for baseline age, sex, age:sex interaction, treatment group, multiple sclerosis disease duration, time to new T2 lesion onset, residual volume of new T2 lesion, baseline T1-Gd^+^ lesion count and baseline non-enhancing total T2 hyperintense lesion volume), a significantly lower nMTR was confirmed in pre-lesion NAWM versus both overall and spatially matched contralateral NAWM at all time points from Week 8 to Week 24 prior to new T2 lesion onset (Fig. 3D and E). Among all covariates evaluated in the multivariate model analysis of the test data set, only younger age and females versus males appeared to be associated with greater pre-lesion NAWM abnormalities.

**Figure 4:**
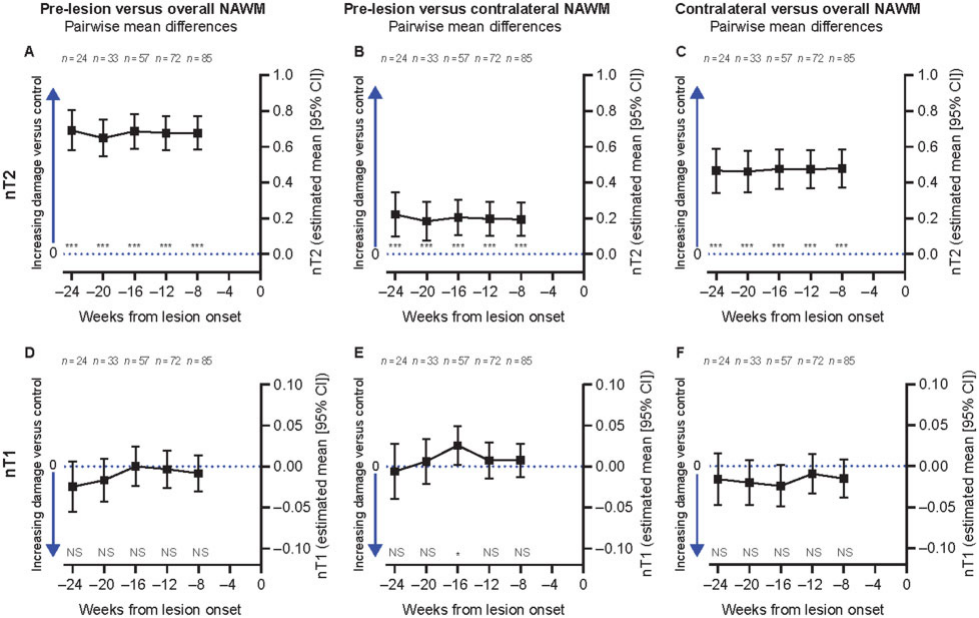
**Pre-lesion NAWM abnormalities are significantly T2 hyperintense while remaining T1 isointense (Test data set).** Significantly greater nT2 intensity in pre-lesion NAWM regions at all time points from Week 8 to Week 24 as compared to both overall NAWM (**A**) and spatially matched contralateral NAWM (**B**); and in spatially matched contralateral NAWM as compared to overall NAWM (**C**). No significant difference in nT1 intensity in pre-lesion NAWM regions as compared to overall NAWM (**D**) and spatially matched contralateral NAWM (**E**); no difference also seen in spatially matched contralateral NAWM as compared to overall NAWM (**F**). NS = not significant. Univariate patient-weighted mixed-effects model analysis. *P < 0.05, **P < 0.01, ***P < 0.001. Similar results were obtained in a multivariate analysis. All tests were based on the least-square means estimates from the weighted mixed-effects models.

Pre-lesion NAWM regions were also observed to have higher nT2 intensity as compared to both overall and spatially matched contralateral NAWM at all time points from Week ‒8 to Week ‒24, prior to new T2 lesion onset in SYNERGY patients with RMS (Fig. 4A and B). In contrast, no significant difference in nT1 signal intensity (Fig. 4D and E) nor in fractional anisotropy (data not shown) was observed at any time point from Week ‒8 to Week ‒24 prior to new T2 lesion onset in pre-lesion NAWM, as compared to either overall and spatially matched contralateral NAWM. Significantly lower nMTR and higher nT2 intensity were also observed in spatially matched contralateral NAWM as compared to overall NAWM (Figs 3C, F and 4C).

### Assessment of NAWM tissue integrity up to 2 years prior to new T2 lesion onset in the validation data set

To validate the findings of the test data set and to explore a longer timeframe, we assessed pre-lesion NAWM for new T2 lesions in patients with RRMS and SPMS from ADVANCE and ASCEND, respectively. Because nMTR was not available for these studies with scanner-matched HCs for normalization, patient-weighted mixed-effects model analysis of pre-lesion NAWM was performed using nT2 intensity.

**Figure 5:**
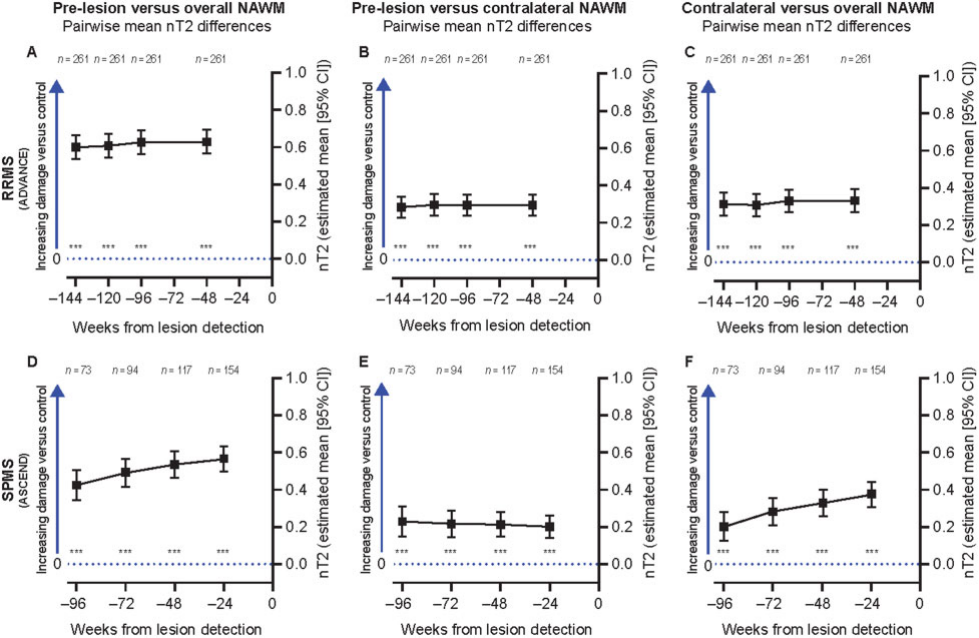
**Pre-lesion NAWM is abnormal at least up to 2 years prior to lesion onset in RRMS and SPMS (validation data sets).** Significantly greater nT2 intensity in pre-lesion NAWM compared to both overall NAWM (**A, D**) and spatially matched contralateral NAWM (**B, E**) at all pre-lesion time points up to 2 years prior to lesion formation in patients with RRMS (**A, B**) and SPMS (**D, E**); greater nT2 intensity was also seen in spatially matched contralateral NAWM compared with overall NAWM in RRMS (**C**) and SPMS (**F**). NS, not significant. Univariate patient-weighted mixed-effects model analysis. *P < 0.05, **P < 0.01, ***P < 0.001. Similar results were obtained in a multivariate analysis. All tests were based on the least-square means estimates from the weighted mixed-effects models. For complete model output, see Supplementary material-3 and Supplementary material-4.

In a univariate analysis, a significantly higher nT2 intensity was observed in pre-lesion NAWM compared to both overall and spatially matched contralateral NAWM at all pre-lesion time points (*P* < 0.001 for all), which extended up to 2 years prior to lesion formation both in patients with RRMS (Fig. 5A and B) and SPMS (Fig. 5D and E). Similar to SYNERGY, nT2 intensity was also significantly higher in spatially matched contralateral NAWM compared with overall NAWM in both ADVANCE RRMS (Fig. 5C) and ASCEND patients with SPMS (Fig. 5F). A multivariate mixed-effects model analysis (adjusted for baseline age, sex, age:sex interaction, treatment group, multiple sclerosis disease duration, time to new T2 lesion onset, residual volume of new T2 lesion, baseline T1-Gd^+^ lesion count and baseline non-enhancing total T2 hyperintense lesion volume) confirmed a significantly higher nT2 intensity in pre-lesion NAWM versus both overall and spatially matched contralateral NAWM at all time points up to 2 years prior to lesion formation in both RRMS and SPMS validation data sets (*P* < 0.001 for all). In contrast with SYNERGY, none of the covariates, including age and sex, were associated with pre-lesion NAWM abnormalities in validation data sets.

The higher nT2 intensities observed in pre-lesion NAWM as well as in spatially matched contralateral NAWM compared to overall NAWM were rather stable over time from 144 to 48 weeks prior to lesion formation in RRMS; a significant increase in nT2 hyperintensity (relative to overall NAWM) was observed over the interval from 96 to 24 weeks prior to lesion formation in patients with SPMS both in pre-lesion and contralateral NAWM (Fig. 5D and F).

### Spatial pattern of NAWM abnormalities and the probabilistic mapping of multiple sclerosis lesions

**Figure 6:**
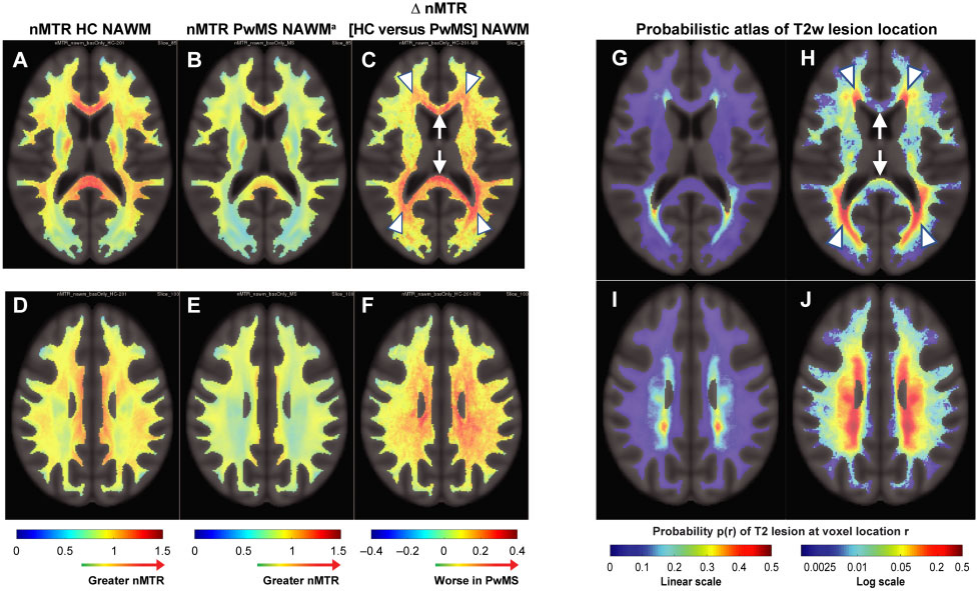
**The spatial pattern of NAWM abnormalities largely recapitulates the mapping of T2 hyperintense multiple sclerosis lesion probability.** These data indicate that the areas of the brain NAWM showing the greatest abnormalities in PwMS mainly involve white matter closest to CSF-filled ventricular spaces (hotter regions in **C, F**). HC acquisition from scanner-matched dummy-run participants (*n* = 110) in all SYNERGY study centres (**A, D**). PwMS (SYNERGY intention-to-treat population, *n* = 419). ^a^PwMS NAWM refers to tissue classified as NAWM at all longitudinal study time points (baseline to Week 72) and at least 2 mm away from any tissue voxel classified T2 hyperintense at any study time point in SYNERGY (**B, E**). In **C and F**, the voxel-wise difference in mean nMTR between HCs and PwMS (baseline scans) is represented; arrows point to the genu and splenium of the corpus callosum and arrowheads to the periventricular regions. In the PwMS group, all longitudinal brain MRIs of the SYNERGY population were used to compute the probabilistic atlas of T2 hyperintense lesion distribution (**G–J**). A 3D video format of Fig. 6 is available as Video 1.

Two-level axial slices (Fig. 6) and complete three-dimensional imaging representations (Videos 1 and 2) showed substantial co-localization of multiple sclerosis-related NAWM abnormalities with regions where T2 hyperintense lesions are more likely to occur. Periventricular regions were characterized by the highest level of multiple sclerosis-related NAWM abnormalities (Fig. 6C, F and Video 1C) and had the highest risk of T2 hyperintense lesions (Fig. 6G–J and Video 1D–E). In contrast, the genu and splenium of the corpus callosum area appeared to have a relatively high multiple sclerosis–related NAWM tissue alteration (Fig. 6C and Video 1C) and a relatively lower risk of T2 hyperintense lesion (Fig. 6G–H and Video 1D–E) compared to periventricular regions. Importantly, absolute mean nMTR also had sizeable variation across anatomical white matter area locations in HCs with highest levels in the corpus callosum. (Fig. 6A and Video 1A). In the corpus callosum of PwMS, despite a lower nMTR compared to HCs, the absolute mean nMTR was higher than in the periventricular areas (Fig. 6B and Video 1B).

**Figure 7:**
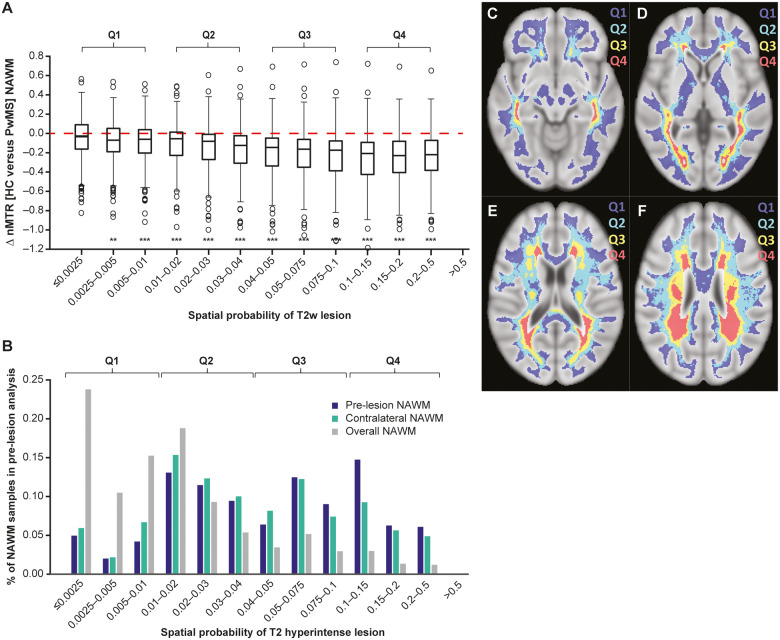
**NAWM abnormalities in PwMS are mostly detected in areas of highest probability of T2 hyperintense lesion formation.** (**A**) Multiple sclerosis–related NAWM abnormalities as measured by nMTR were significantly more severe in NAWM areas of higher probability of T2 hyperintense lesions (SYNERGY intention-to-treat baseline scans). The test in panel (**A**) is a likelihood ratio test comparing two models, one of which models the interaction between bins and subject population and the effect of bins is modelled as continuous using a linear term and the other does not model the interaction effect. (**B**) Pre-lesion NAWM abnormalities and spatially matched contralateral NAWM analysed in SYNERGY occurred preferentially in areas with higher probability of T2 hyperintense lesions compared to overall NAWM. The test in panel (**B**) is also a likelihood ratio test comparing two models, as in panel (**A**), but the effect of bin is modelled as discrete. PwMS NAWM refers to tissue classified as NAWM at all longitudinal study time points (baseline to Week 72) in SYNERGY (intention-to-treat). Panels **C–F** represent axial brain slices of the anatomical distribution of Q1, Q2, Q3 and Q4 categories of spatial probabilities of T2 hyperintense lesion also represented in 3D video format in Video 3E). **P < 0.01 and ***P < 0.001 versus first bin of NAWM tissue where probability p(*r*) of T2 hyperintense lesion at each voxel location *r* is minimal and <0.0025. Q1–Q4 are in reference to probability categories depicted in Video 3.

Subdividing the multiple sclerosis lesion probability atlas (shown in Fig. 6G‒J and Video 1D‒E) into discrete bins shows bands of increasing probabilities of multiple sclerosis lesion in white matter tissue distributed from juxtacortical to periventricular regions (Fig. 7A, C‒F and Video 3). Multiple sclerosis–related NAWM abnormalities, as measured by nMTR difference between PwMS and HCs, were statistically significant in NAWM areas of higher probability of T2 hyperintense lesions but not in areas where the probability of T2 hyperintense lesions was lowest (as illustrated by asterisks in Fig. 7A). When treated as continuous variables, the NAWM abnormality and probability of lesion formation within the 12 discrete bins continuous variable were significantly correlated (*P* < 0.001).

A frequency distribution representation (Fig. 7B) demonstrated that pre-lesion and spatially matched contralateral areas of NAWM analysed in SYNERGY were relatively more distributed in areas with higher probability of T2 hyperintense lesions in comparison to overall NAWM (Fig. 7B). When further adjusting for multiple sclerosis T2 hyperintense lesion probabilities as a continuous variable in specific ROIs, the multivariate mixed-effects model analysis confirmed significant nMTR abnormalities in pre-lesion NAWM versus both overall and spatially matched contralateral NAWM.

## Discussion

Analysis of pre-lesion NAWM in three large multiple sclerosis clinical trials demonstrated that NAWM tissue state abnormalities may be detected as early as 144 weeks (the longest interval available) prior to lesion formation. In the test data set, a lower nMTR and higher nT2 intensity was observed in pre-lesion NAWM up to 24 weeks prior to the appearance of new lesions compared with both overall and spatially matched contralateral NAWM. In validation cohorts, relative T2 hyperintensity was already present approximately 2 years prior to lesion formation, compared to both overall and spatially matched contralateral NAWM. The topologic pattern of multiple sclerosis–related NAWM abnormalities, as measured by nMTR intensity, largely recapitulated the probabilistic atlas of multiple sclerosis T2 hyperintense lesion location coalescing predominantly in periventricular regions. Although NAWM abnormalities are more severe in NAWM areas with higher probability of lesion, the significant differences between abnormalities observed in pre-lesion compared to matched contralateral NAWM suggest that precursory abnormalities in pre-lesion NAWM are attributable at least in part to the specific subsequent event of ‘new’ lesion formation.

The finding of a reduced MTR as a pre-lesion abnormality in NAWM is consistent with previous observations,^16–19^ albeit more robust (confirmed by multivariate analysis) on account of data derived from a larger cohort with a longer pre-lesion assessment duration. In the SYNERGY dataset, younger age and female sex were covariates associated with greater pre-lesion NAWM abnormalities, although these findings were not corroborated in the larger ASCEND and ADVANCE validation datasets. Importantly, multiple sclerosis disease duration, time since new T2 lesion onset, residual volume of new T2 lesion, baseline T1-Gd^+^ lesion counts and baseline non-enhancing T2 hyperintense lesion volume had no effect on pre-lesion NAWM abnormalities in the multivariate model. This suggests that pre-lesion NAWM abnormalities may represent either an intrinsic property of the tissue state in areas at higher risk of lesion formation that may be independent of multiple sclerosis disease stage and activity, and/or that chronic latent white matter abnormalities develop years before ‘lesion’ formation.

In both the test and validation cohorts included in this study, a relative T2 hyperintensity was observed prior to lesion formation, which is consistent with a study that was conducted in a smaller population with active multiple sclerosis over a shorter duration.^25^ Although the T2 hyperintensity in the pre-lesion ROIs was stable over time in the RMS population of SYNERGY and the RRMS population of ADVANCE, there was an incremental increase over time prior to lesion formation in T2 hyperintensity for the SPMS population in ASCEND, both in pre-lesion and spatially matched contralateral NAWM. This suggests that pre-lesion and contralateral NAWM in patients with more advanced SPMS may manifest superimposed pathology that worsens over time.

The significant reduction in nMTR and/or relative T2 hyperintensity in spatially matched contralateral NAWM compared with overall NAWM was consistent in the different multiple sclerosis subtypes in the three trial populations analysed here. Alterations in contralateral NAWM have been observed in a previous study investigating whether changes in diffusion coefficients occur prior to lesion formation in multiple sclerosis.^21^ In this referenced study, changes in homologous contralateral NAWM were detected after lesion formation and only observed once the contralateral new acute lesion enhanced and were attributed to potential damage to trans-hemispheric projection fibres traversing the acute lesion, with subsequent Wallerian degeneration. This hypothesis of the role of trans-hemispheric connectivity in the spreading of pathology was complemented by another report of reductions in N-acetyl aspartate after lesion formation in regions contralateral to demyelinating lesions,^38^ the latter likely due to a reduction in the integrity of neurons.^39^ In this study, we demonstrated the existence of longstanding precursory NAWM abnormalities in regions contralateral to where lesions will form, which were located in areas of higher lesion probabilities, as compared to overall NAWM. The hypothesis that a causal ‘factor’ underlying precursory NAWM abnormalities spatially associated with the probability of subsequent lesion formation may be tracking to the opposite hemisphere remains to be further explored.

The evidence that NAWM abnormalities in PwMS are spatially associated with areas of higher probability of lesion formation may indicate a spatially regulated causality relationship between latent white matter tissue alteration and the risk of lesion formation. An exception to this pattern was observed in the corpus callosum which appeared to be an area of important NAWM nMTR difference in PwMS versus HCs but associated with a relatively low risk of lesion formation. An explanation could be that what is most associated with the risk of lesion formation is the spatial distribution of NAWM tissue damage measured by lower absolute nMTR levels rather than the difference versus HCs in absolute nMTR levels. The absolute nMTR levels remained relatively high in the corpus callosum of PwMS compared to lowest absolute nMTR values observed in periventricular areas where lesions are most likely to form.

In the absence of post-mortem correlates, the precise pathology that may underlie the observed MRI alterations in pre-lesion NAWM tissue remains unclear. In this study, pre-lesion NAWM tissue abnormalities were identified by MTR and T2-weighted imaging but were not associated with alterations in T1 intensities or fractional anisotropy in diffusion tensor imaging. Regardless, it is possible that subtle myelin pathology, microglial activation, axonal degeneration or oligodendrocyte apoptosis could be contributing factors to the MTR reductions and relative T2 hyperintensity that were observed.^15^^,^^40–45^ This could imply that multiple sclerosis lesions may occur as a consequence of primary and longstanding functional, biochemical and/or structural disruption of myelin-axon homeostatic processes that may precede inflammatory autoimmune attacks of the disease.^46–48^

Biochemically altered myelin without demyelination may also be at play, as sensitive spectroscopic techniques using the lipophilic dye Nile red have identified apparently intact yet altered myelin in the multiple sclerosis brain as well as in pre-lesion tissues of mouse white matter following sub-demyelinating cuprizone intoxication.^49^ Intriguingly, abnormalities of myelin dielectric constant measured by the Nile Red method also revealed a pathological gradient in human NAWM, maximal adjacent to the ventricular surface, in accord with the pre-lesion abnormalities detected by MRI in the current study.^49^ Recent works have also suggested that an initial disintegration of myelin, resulting in the release of post-translationally modified myelin antigens, might elicit a secondary immune attack in multiple sclerosis.^50^

Alternative possibilities are that these pre-lesion changes may represent either expanding but microscopic lesions,^17^^,^^40^^,^^44^^,^^51^ Wallerian degeneration,^52^ astrocytic proliferation^27^ or changes in water content that would be consistent with a very slowly evolving perivascular inflammatory process of peripheral origin. Pathological specimens were not available to confirm these competing hypotheses. Future high-resolution MRI work may be helpful to quantitatively elucidate whether the spatial distribution pattern of small vessel venous density in HCs and PwMS^53^^,^^54^ may recapitulate that of multiple sclerosis–related MRI abnormalities in NAWM mapped to the probabilistic atlas of multiple sclerosis lesions with a preponderance in anterior and posterior periventricular regions.

There are a number of strengths to this current study, which include the large sample size and long duration of backward follow-up prior to lesion formation. Furthermore, the findings from the test cohort were replicated in the validation cohorts, which contained populations of both relapsing and progressive forms of multiple sclerosis. From a methodological perspective, all automatic identifications of new T2 lesions were manually reviewed for false detection and strict criteria were utilized to characterize pre-lesion and contralateral NAWM. These criteria were pre-determined to avoid inclusion of regions subject to influences from surrounding pre-existing lesions. The use of three pairwise differences including overall NAWM enabled characterization of abnormalities in both pre-lesion and contralateral NAWM. Limitations of this study included the lack of nMTR acquisitions for the larger ASCEND and ADVANCE trials to validate the nMTR findings from SYNERGY.

In summary, we have provided evidence from three large clinical trial cohorts including long-term follow-up that longstanding abnormalities, including reduced nMTR and relative T2 hyperintensity, are observed prior to lesion development and that, in general, NAWM abnormalities are more pronounced at locations with high likelihood of developing subsequent multiple sclerosis lesions. Our findings are consistent with the concept of a precursory multiple sclerosis lesion, where foci of subtle biochemical and/or structural alteration of white matter predates the more typical lesion by many years, which may have implications for our understanding of the underlying pathophysiology and disease course of multiple sclerosis. Spatially regulated measures of NAWM abnormalities in PwMS may also be used as novel anchoring traits for the identification and validation of candidate molecular and cellular targets for drug development strategies targeting the underlying pathobiology of multiple sclerosis.

Crucial areas for further investigation include prospective experiments to determine causal inference as to whether MRI patterns of NAWM abnormalities may predict when and where lesions may ultimately arise in PwMS. Addressing this seminal question may elucidate whether multiple sclerosis lesion formation is primarily an outside-in stochastic phenomenon or, alternatively, may correspond to initial biochemical modification of the CNS parenchyma, predisposed to occur in specific regions, notably in proximity to CSF where intrinsic, possibly cytodegenerative cues may reside, leading to secondary autoimmune inflammatory attacks.

## Supplementary material

Supplementary material is available at *Brain Communications* online.

## Supplementary Material

fcab176_Supplementary_Data

## Abbreviations

HC =: healthy control
ICBM =: International Consortium for Brain Mapping
MTR =: magnetization transfer ratio
NAWM =: normal appearing white matter
nMTR =: normalized MTR
nT1 =: normalized T1-weighted
nT2 =: normalized T2-weighted
PwMS =: persons with multiple sclerosis
RMS =: relapsing multiple sclerosis
ROI =: region of interest
RRMS =: relapsing-remitting multiple sclerosis
SPMS =: secondary progressive multiple sclerosis
