# Supplementary material for: Abnormalities in normal-appearing white matter from which multiple sclerosis lesions arise
Source: Brain Commun. 2021 Aug 10;3(3):fcab176. doi: 10.1093/braincomms/fcab176 (PMC8453433; doi:10.1093/braincomms/fcab176)
Supplement: fcab176_Supplementary_Data [file fcab176_Supplementary_Data.zip › suppl_data/Supplementary Material.pdf]

SUPPLEMENTARY MATERIAL

Abnormalities in normal-appearing white matter from which multiple sclerosis lesions arise

Colm Elliott,[1] Parya Momayyezsiahkal,[1-2] Douglas L. Arnold,[1-2] Dawei Liu,[3] Jun Ke,[3] Li Zhu,[3] Bing Zhu,[3] Ilena C. George,[4] Daniel P. Bradley,[3] Elizabeth Fisher,[3] Ellen Cahir-McFarland,[3*] Peter K. Stys,[5] Jeroen J.G. Geurts,[6] Nathalie Franchimont,[3] Arie Gafson,[3] Shibeshih Belachew[3]

[1]*NeuroRx Research, Montreal, QC, Canada*
[2]*McGill University, Montreal, QC, Canada*
[3]*Biogen, Cambridge, MA, USA*
[4]*Massachusetts General Hospital Department of Neurology, Boston, MA, USA*
[5]*Department of Clinical Neurosciences, Hotchkiss Brain Institute, University of Calgary Cumming School of Medicine, Calgary, AB, Canada*
[6]*Amsterdam UMC, Vrije Universiteit, Department of Anatomy and Neurosciences, Amsterdam Neuroscience, Amsterdam, the Netherlands*

Contents

Abbreviations 2

Supplementary Materials

Supplementary Material 1. MRI protocol – SYNERGY 3

Supplementary Material 2. Composite data for Figure 3 5

Supplementary Material 3. Composite data for Figure 5A-C 11

Supplementary Material 4. Composite data for Figure 5D-F 16

Supplementary Table

Supplementary Table 21

Supplementary Figure

Supplementary Figure. Example of the patient-level segmentation of ROIs for the analysis of pre-lesion, overall, and spatially matched contralateral NAWM prior to new 22

Abbreviations

AGE:SEXM=age:sex (male) interaction
CGDBL=baseline total T1 gadolinium-enhancing lesion count
CI=confidence interval
DF=degrees of freedom
DTI= diffusion tensor imaging
EPI=echo-planar imaging
Gd^+=gadolinium-enhancing
ITT=intent-to-treat
LS=least square
lsmean=least square mean
MRI=magnetic resonance imaging
MS=multiple sclerosis
MSONSET=time since MS symptom onset
MT=magnetization transfer
MTR=magnetization transfer ratio
NAWM=normal appearing white matter
nMTR=normalized magnetization transfer ratio
prob.diff=difference in probabilities of T2w lesion location between pre-lesion NAWM regions of interest (ROI) and spatially matched contralateral NAWM ROI
PwMS=persons with multiple sclerosis
RESIDVOL=new T2 lesion volume remaining at end of study (residual new T2) which were used as pre-lesion ROI
RRMS=relapsing-remitting multiple sclerosis
RMS=relapsing multiple sclerosis
ROI=region of interest
SE=Spin Echo (ie, MRI context)
SE=standard error (ie, statistical context)
SEXM=male sex
SPMS=secondary progressive multiple sclerosis
T1w=T1 weighted
T2w=T2 weighted
T2VBL=baseline total non-enhancing T2 lesion volume
TE=echo time (basic pulse sequence parameter)
TR=repetition time (basic pulse sequence parameter)
tRel=time relative to lesion detection
tRelw-X= time relative to lesion detection at Week 'X'

Supplementary Materials

Supplementary Material 1. MRI protocol - SYNERGY

All brain MRI sequences used in this work were acquired according to the study protocol. Each scanner was approved with one set of specific parameters, included in the ranges provided below.

Pre-gadolinium injection:
Sagittal T1w (3D Spoiled gradient echo, TR = 15–22 ms; TE = 6–10 ms; flip angle = 27–30°)
Axial T2w (2D Fast Spin Echo, TR = 4500–6200 ms; TE = 66–91 ms; ETL = 7–11)
Axial MT OFF and MT ON (3D Spoiled gradient echo, TR = 32–62 ms; TE = 5–11 ms; flip angle = 10–15°; MT ON has in addition an Off-resonance magnetization transfer pulse)
Axial DTI sequence (2D SE EPI sequence with a diffusion gradient, TR = 9800–16000 ms; TE = 90–132 ms; b-values = 0, 1000; 25–36 diffusion directions)
Axial T1w (3D Spoiled gradient echo, TR = 28–30 ms; TE = 5–11 ms; flip angle = 27–30°)
Post-gadolinium injection (0.1 mmol/kg): Axial T1w (3D Spoiled gradient echo, TR = 28–30 ms; TE = 5–11 ms; flip angle = 27–30°) 10 minutes after the end of the injection

Axial T2w, MT Off, MT On, and T1w pre/post gadolinium sequences had 60 slices with a 3-mm thickness and 0.98*0.98 in-plane resolution.

Sagittal T1w had 132–160 slices with a 1.2-mm thickness and a 1.2*1.2 in-plane resolution.

Axial DTI had 58–60 slices with a 2.5-mm thickness and a 2.5*2.5mm in-plane resolution.

MRI protocol - ASCEND

All brain MRI sequences were acquired according to the study protocol. Each scanner was approved with one set of specific parameters, included in the ranges provided below.

Pre-gadolinium injection:
Axial T1w (3D Spoiled gradient echo, TR = 28–35 ms; TE = 4–11 ms; flip angle = 27–30°)
Axial T2w (2D Fast Spin Echo, TR = 4000–7400 ms; TE = 58–95ms)

Axial T1w and T2w had 60 slices with a 3-mm thickness and 0.98*0.98 in-plane resolution.

MRI protocol - ADVANCE

All brain MRI sequences were acquired according to the study protocol. Each scanner was approved with one set of specific parameters, included in the ranges provided below.

Pre-gadolinium injection:

Axial T1w (3D Spoiled gradient echo, TR = 28–35 ms; TE = 4–11 ms; flip angle = 27–30°)

Axial T2w (2D Fast Spin Echo, TR = 4000–7720 ms; TE = 56–93ms)

Axial T1w and T2w had 60 slices with a 3-mm thickness and 0.98*0.98 in-plane resolution.

MRI standardization procedure

Conventional MRI was standardized across scanners to achieve consistent contrast for each of the conventional sequences. MTR was standardised as previously described (Brown *et al.*, 2013).

Reference

Brown RA, Narayanan S, Arnold DL. Segmentation of magnetization transfer ratio lesions for longitudinal analysis of demyelination and remyelination in multiple sclerosis. Neuroimage 2013; 66: 103–9.

Supplementary Material 2. Composite data for Figure 3

1. Univariate analysis – Pairwise nMTR intensity differences in SYNERGY trial population (Figure 3A-C)

1) Pre-lesion vs Contralateral NAWM

Mixed model estimate

	Value	Std.Error	DF	t.value	p.value
(Intercept)	-0.068	0.017	301	-3.979	0.000
tRelw-12	0.010	0.017	301	0.545	0.586
tRelw-16	-0.032	0.020	301	-1.555	0.121
tRelw-20	-0.030	0.025	301	-1.214	0.226
tRelw-24	0.010	0.033	301	0.318	0.751

LS means estimate

tRel	lsmean	SE	df	t.ratio	p.value
w-8	-0.068	0.017	83	-3.979	0.000
w-12	-0.058	0.019	83	-3.073	0.003
w-16	-0.099	0.021	83	-4.656	0.000
w-20	-0.098	0.026	83	-3.797	0.000
w-24	-0.057	0.033	83	-1.725	0.088

Effect size estimate -- omega_squared

Parameter	Omega2_partial	CI	CI_low	CI_high
tRel	0.005	0.9	0.000	0.012

Effect size estimate -- eta_squared

Parameter	Eta2_partial	CI	CI_low	CI_high
tRel	0.018	0.9	0.000	0.039

2) Contralateral versus Overall NAWM

Mixed model estimate

	Value	Std.Error	DF	t.value	p.value
(Intercept)	-0.059	0.013	301	-4.424	0.000
tRelw-12	-0.002	0.011	301	-0.204	0.838
tRelw-16	0.025	0.013	301	1.888	0.060
tRelw-20	0.022	0.016	301	1.350	0.178
tRelw-24	0.021	0.021	301	0.995	0.321

LS means estimate

tRel	lsmean	SE	df	t.ratio	p.value
w-8	-0.059	0.013	83	-4.424	0.000
w-12	-0.061	0.014	83	-4.255	0.000
w-16	-0.034	0.016	83	-2.166	0.033
w-20	-0.037	0.018	83	-2.027	0.046
w-24	-0.038	0.023	83	-1.666	0.099

Effect size estimate -- omega_squared

Parameter	Omega2_partial	CI	CI_low	CI_high
tRel	0.006	0.9	0.000	0.015

Effect size estimate -- eta_squared

Parameter	Eta2_partial	CI	CI_low	CI_high
tRel	0.020	0.9	0.000	0.041

3) Pre-lesion vs Overall NAWM

Mixed model estimate

	Value	Std.Error	DF	t.value	p.value
(Intercept)	-0.125	0.018	301	-7.051	0.000
tRelw-12	0.009	0.012	301	0.771	0.441
tRelw-16	-0.004	0.014	301	-0.281	0.779
tRelw-20	-0.004	0.018	301	-0.217	0.828
tRelw-24	0.038	0.023	301	1.650	0.100

LS means estimate

tRel	lsmean	SE	df	t.ratio	p.value
w-8	-0.125	0.018	83	-7.051	0.000
w-12	-0.115	0.019	83	-6.197	0.000
w-16	-0.129	0.020	83	-6.488	0.000
w-20	-0.129	0.022	83	-5.752	0.000
w-24	-0.087	0.027	83	-3.249	0.002

Effect size estimate -- omega_squared

Parameter	Omega2_partial	CI	CI_low	CI_high
tRel	0.001	0.9	0.000	0.000

Effect size estimate -- eta_squared

Parameter	Eta2_partial	CI	CI_low	CI_high
tRel	0.014	0.9	0.000	0.031

2. Multivariate analysis – Pairwise nMTR intensity differences in SYNERGY trial population (Figure 3D-F)

1) Pre-lesion vs Contralateral NAWM

Mixed model estimate

	Value	Std.Error	DF	t.value	p.value
(Intercept)	-0.308	0.158	301	-1.945	0.053
tRelw-12	0.009	0.018	301	0.540	0.589
tRelw-16	-0.030	0.020	301	-1.468	0.143
tRelw-20	-0.029	0.025	301	-1.140	0.255
tRelw-24	0.012	0.033	301	0.375	0.708
AGE	0.008	0.003	73	2.484	0.015
SEXM	0.322	0.154	73	2.098	0.039
MSONSET	-0.005	0.003	73	-1.547	0.126
log10.RESIDVOL	0.014	0.051	94	0.266	0.791
CGDBL	-0.002	0.002	73	-1.074	0.286
T2VBL	0.001	0.001	73	0.467	0.642
prob.diff	-1.336	0.380	94	-3.518	0.001
ARMBIIB033 3 mg/kg + Avonex	-0.012	0.058	73	-0.199	0.843
ARMBIIB033 10 mg/kg + Avonex	-0.016	0.046	73	-0.354	0.725
ARMBIIB033 30 mg/kg + Avonex	-0.041	0.045	73	-0.912	0.365
ARMBIIB033 100 mg/kg + Avonex	-0.007	0.049	73	-0.135	0.893
AGE:SEXM	-0.009	0.004	73	-2.030	0.046

LS means estimate

tRel	lsmean	SE	df	t.ratio	p.value
w-8	-0.077	0.019	73	-4.066	0.000
w-12	-0.067	0.020	73	-3.333	0.001
w-16	-0.107	0.022	73	-4.786	0.000
w-20	-0.105	0.027	73	-3.945	0.000
w-24	-0.065	0.034	73	-1.895	0.062

Effect size estimate -- omega_squared

Parameter	Omega2_partial	CI	CI_low	CI_high
tRel	0.005	0.9	0.000	0.011
AGE	-0.003	0.9	0.000	0.000
SEX	-0.013	0.9	0.000	0.000
MSONSET	-0.007	0.9	0.000	0.000
log10.RESIDVOL	-0.010	0.9	0.000	0.000
CGDBL	-0.009	0.9	0.000	0.000
T2VBL	-0.013	0.9	0.000	0.000
prob.diff	0.123	0.9	0.038	0.232
ARM	-0.037	0.9	0.000	0.000
AGE:SEX	0.040	0.9	0.000	0.137

Effect size estimate -- eta_squared

Parameter	Eta2_partial	CI	CI_low	CI_high
tRel	0.018	0.9	0.000	0.039
AGE	0.011	0.9	0.000	0.081
SEX	0.000	0.9	0.000	0.024
MSONSET	0.006	0.9	0.000	0.068
log10.RESIDVOL	0.000	0.9	0.000	0.007
CGDBL	0.004	0.9	0.000	0.060
T2VBL	0.000	0.9	0.000	0.000
prob.diff	0.133	0.9	0.044	0.244
ARM	0.017	0.9	0.000	0.031
AGE:SEX	0.053	0.9	0.001	0.157

2) Contralateral versus Overall NAWM

Mixed model estimate

	Value	Std.Error	DF	t.value	p.value
(Intercept)	0.283	0.127	301	2.223	0.027
tRelw-12	-0.003	0.011	301	-0.221	0.825
tRelw-16	0.025	0.013	301	1.863	0.063
tRelw-20	0.021	0.016	301	1.311	0.191
tRelw-24	0.019	0.021	301	0.895	0.371
AGE	-0.001	0.002	73	-0.328	0.744
SEXM	-0.033	0.123	73	-0.265	0.792
MSONSET	0.001	0.003	73	0.465	0.644
log10.RESIDVOL	-0.161	0.041	94	-3.893	0.000
CGDBL	0.001	0.002	73	0.590	0.557
T2VBL	0.001	0.001	73	0.636	0.527
prob.diff	0.302	0.312	94	0.968	0.336
ARMBIIB033 3 mg/kg + Avonex	0.067	0.048	73	1.400	0.166
ARMBIIB033 10 mg/kg + Avonex	0.028	0.037	73	0.760	0.450
ARMBIIB033 30 mg/kg + Avonex	-0.011	0.037	73	-0.313	0.755
ARMBIIB033 100 mg/kg + Avonex	0.015	0.039	73	0.392	0.697
AGE:SEXM	-0.001	0.003	73	-0.162	0.872

LS means estimate

tRel	lsmean	SE	df	t.ratio	p.value
w-8	-0.059	0.015	73	-4.026	0.000
w-12	-0.062	0.015	73	-3.992	0.000
w-16	-0.035	0.017	73	-2.075	0.041
w-20	-0.038	0.019	73	-1.979	0.052
w-24	-0.040	0.024	73	-1.710	0.092

Effect size estimate -- omega_squared

Parameter	Omega2_partial	CI	CI_low	CI_high
tRel	0.006	0.9	0.000	0.014
AGE	-0.013	0.9	0.000	0.000
SEX	0.036	0.9	0.000	0.130
MSONSET	-0.007	0.9	0.000	0.000
log10.RESIDVOL	0.130	0.9	0.042	0.240
CGDBL	-0.009	0.9	0.000	0.000

Parameter	Omega2_partial	CI	CI_low	CI_high
T2VBL	-0.007	0.9	0.000	0.000
prob.diff	-0.002	0.9	0.000	0.000
ARM	-0.016	0.9	0.000	0.000
AGE:SEX	-0.013	0.9	0.000	0.000

Effect size estimate -- eta_squared

Parameter	Eta2_partial	CI	CI_low	CI_high
tRel	0.019	0.9	0.000	0.040
AGE	0.001	0.9	0.000	0.002
SEX	0.049	0.9	0.000	0.151
MSONSET	0.007	0.9	0.000	0.069
log10.RESIDVOL	0.140	0.9	0.049	0.251
CGDBL	0.004	0.9	0.000	0.061
T2VBL	0.006	0.9	0.000	0.067
prob.diff	0.009	0.9	0.000	0.064
ARM	0.036	0.9	0.000	0.083
AGE:SEX	0.000	0.9	0.000	0.026

3) Pre-lesion vs Overall NAWM

Mixed model estimate

	Value	Std.Error	DF	t.value	p.value
(Intercept)	0.002	0.157	301	0.012	0.990
tRelw-12	0.009	0.012	301	0.746	0.456
tRelw-16	-0.004	0.014	301	-0.260	0.795
tRelw-20	-0.004	0.018	301	-0.217	0.828
tRelw-24	0.037	0.023	301	1.604	0.110
AGE	0.007	0.003	73	2.138	0.036
SEXM	0.266	0.152	73	1.753	0.084
MSONSET	-0.004	0.003	73	-1.278	0.205
log10.RESIDVOL	-0.151	0.051	94	-2.961	0.004
CGDBL	-0.001	0.002	73	-0.642	0.523
T2VBL	0.001	0.001	73	1.041	0.301
prob.diff	-0.966	0.388	94	-2.490	0.015
ARMBIIB033 3 mg/kg + Avonex	0.055	0.060	73	0.922	0.360
ARMBIIB033 10 mg/kg + Avonex	0.013	0.046	73	0.284	0.777
ARMBIIB033 30 mg/kg + Avonex	-0.055	0.046	73	-1.182	0.241
ARMBIIB033 100 mg/kg + Avonex	0.000	0.048	73	0.000	1.000
AGE:SEXM	-0.009	0.004	73	-2.051	0.044

LS means estimate

tRel	lsmean	SE	df	t.ratio	p.value
w-8	-0.134	0.018	73	-7.418	0.000
w-12	-0.125	0.019	73	-6.638	0.000
w-16	-0.138	0.020	73	-6.894	0.000
w-20	-0.138	0.023	73	-6.130	0.000
w-24	-0.098	0.027	73	-3.619	0.001

Effect size estimate -- omega_squared

Parameter	Omega2_partial	CI	CI_low	CI_high
tRel	0.000	0.9	0.000	0.000
AGE	-0.008	0.9	0.000	0.000
SEX	0.020	0.9	0.000	0.101
MSONSET	-0.012	0.9	0.000	0.000
log10.RESIDVOL	0.090	0.9	0.019	0.192
CGDBL	-0.013	0.9	0.000	0.000
T2VBL	-0.007	0.9	0.000	0.000
prob.diff	0.065	0.9	0.008	0.159
ARM	-0.001	0.9	0.000	0.000
AGE:SEX	0.041	0.9	0.000	0.138

Effect size estimate -- eta_squared

Parameter	Eta2_partial	CI	CI_low	CI_high
tRel	0.014	0.9	0.000	0.030
AGE	0.005	0.9	0.000	0.064
SEX	0.033	0.9	0.000	0.126
MSONSET	0.001	0.9	0.000	0.043
log10.RESIDVOL	0.101	0.9	0.025	0.205
CGDBL	0.001	0.9	0.000	0.033
T2VBL	0.007	0.9	0.000	0.070
prob.diff	0.075	0.9	0.012	0.173
ARM	0.051	0.9	0.000	0.112
AGE:SEX	0.054	0.9	0.001	0.159

Supplementary Material 3. Composite data for Figure 5A-C

1. Univariate analysis – Pairwise nT2 intensity differences in ADVANCE RRMS trial population

1) Pre-lesion vs Contralateral NAWM

Mixed model estimate

	Value	Std.Error	DF	t.value	p.value
(Intercept)	0.294	0.029	1881	10.101	0.000
tRelw-96	-0.000	0.018	1881	-0.026	0.979
tRelw-120	0.002	0.018	1881	0.097	0.923
tRelw-144	-0.011	0.018	1881	-0.593	0.553

LS means estimate

tRel	lsmean	SE	df	t.ratio	p.value
w-48	0.294	0.029	260	10.101	0.000
w-96	0.293	0.029	260	10.085	0.000
w-120	0.295	0.029	260	10.161	0.000
w-144	0.283	0.029	260	9.734	0.000

Effect size estimate -- omega_squared

Parameter	Omega2_partial	CI	CI_low	CI_high
tRel	-0.001	0.9	0.000	0.000

Effect size estimate -- eta_squared

Parameter	Eta2_partial	CI	CI_low	CI_high
tRel	0.000	0.9	0.000	0.001

2) Contralateral versus Overall NAWM

Mixed model estimate

	Value	Std.Error	DF	t.value	p.value
(Intercept)	0.331	0.031	1881	10.644	0.000
tRelw-96	-0.001	0.014	1881	-0.094	0.925
tRelw-120	-0.023	0.014	1881	-1.706	0.088
tRelw-144	-0.019	0.014	1881	-1.352	0.177

LS means estimate

tRel	lsmean	SE	df	t.ratio	p.value
w-48	0.331	0.031	260	10.644	0.000
w-96	0.330	0.031	260	10.603	0.000
w-120	0.308	0.031	260	9.893	0.000
w-144	0.313	0.031	260	10.049	0.000

Effect size estimate -- omega_squared

Parameter	Omega2_partial	CI	CI_low	CI_high
tRel	0.001	0.9	0.000	0.003

Effect size estimate -- eta_squared

Parameter	Eta2_partial	CI	CI_low	CI_high
tRel	0.002	0.9	0.000	0.006

3) Pre-lesion vs Overall NAWM

Mixed model estimate

	Value	Std.Error	DF	t.value	p.value
(Intercept)	0.629	0.032	1881	19.466	0.000
tRelw-96	-0.002	0.015	1881	-0.119	0.905
tRelw-120	-0.022	0.015	1881	-1.470	0.142
tRelw-144	-0.029	0.015	1881	-1.982	0.048

LS means estimate

tRel	lsmean	SE	df	t.ratio	p.value
w-48	0.629	0.032	260	19.466	0.000
w-96	0.627	0.032	260	19.412	0.000
w-120	0.607	0.032	260	18.796	0.000
w-144	0.599	0.032	260	18.562	0.000

Effect size estimate -- omega_squared

Parameter	Omega2_partial	CI	CI_low	CI_high
tRel	0.001	0.9	0.000	0.004

Effect size estimate -- eta_squared

Parameter	Eta2_partial	CI	CI_low	CI_high
tRel	0.003	0.9	0.000	0.007

2. Multivariate analysis – Pairwise nT2 intensity differences in ADVANCE RRMS trial population

1) Pre-lesion vs Contralateral NAWM

Mixed model estimate

	Value	Std.Error	DF	t.value	p.value
(Intercept)	0.062	0.197	1881	0.314	0.754
tRelw-96	-0.000	0.018	1881	-0.026	0.979
tRelw-120	0.002	0.018	1881	0.097	0.923
tRelw-144	-0.011	0.018	1881	-0.593	0.553
AGE	0.006	0.004	253	1.686	0.093
SEXM	0.131	0.209	253	0.627	0.531
MSONSET	-0.002	0.006	253	-0.348	0.728
log10.RESIDVOL	0.060	0.071	366	0.837	0.403
ARMBIIB017 125 mcg every 2 weeks	-0.139	0.100	253	-1.393	0.165
ARMBIIB017 125 mcg every 4 weeks	-0.066	0.089	253	-0.742	0.459
ARMPlacebo -> BIIB017 125 mcg every 4 weeks	0.004	0.100	253	0.042	0.966
AGE:SEXM	-0.006	0.006	253	-0.895	0.372

LS means estimate

tRel	lsmean	SE	df	t.ratio	p.value
w-48	0.291	0.035	253	8.300	0.000
w-96	0.290	0.035	253	8.287	0.000
w-120	0.293	0.035	253	8.349	0.000
w-144	0.280	0.035	253	7.996	0.000

Effect size estimate -- omega_squared

Parameter	Omega2_partial	CI	CI_low	CI_high
tRel	-0.001	0.9	0.000	0.000
AGE	0.006	0.9	0.000	0.031
SEX	0.000	0.9	0.000	0.013
MSONSET	-0.004	0.9	0.000	0.000
log10.RESIDVOL	-0.001	0.9	0.000	0.000
ARM	0.001	0.9	0.000	0.000
AGE:SEX	-0.001	0.9	0.000	0.000

Effect size estimate -- eta_squared

Parameter	Eta2_partial	CI	CI_low	CI_high
tRel	0.000	0.9	0.000	0.001
AGE	0.010	0.9	0.000	0.039
SEX	0.004	0.9	0.000	0.028
MSONSET	0.000	0.9	0.000	0.010
log10.RESIDVOL	0.001	0.9	0.000	0.014
ARM	0.013	0.9	0.000	0.034
AGE:SEX	0.003	0.9	0.000	0.025

2) Contralateral versus Overall NAWM

Mixed model estimate

	Value	Std.Error	DF	t.value	p.value
(Intercept)	-0.147	0.211	1881	-0.697	0.486
tRelw-96	-0.001	0.014	1881	-0.094	0.925
tRelw-120	-0.023	0.014	1881	-1.706	0.088
tRelw-144	-0.019	0.014	1881	-1.352	0.177
AGE	-0.000	0.004	253	-0.101	0.920
SEXM	0.052	0.231	253	0.224	0.823
MSONSET	0.000	0.007	253	0.056	0.955
log10.RESIDVOL	0.191	0.075	366	2.541	0.011
ARMBIIB017 125 mcg every 2 weeks	0.251	0.108	253	2.324	0.021
ARMBIIB017 125 mcg every 4 weeks	0.038	0.098	253	0.388	0.698
ARMPlacebo -> BIIB017 125 mcg every 4 weeks	0.161	0.109	253	1.467	0.144
AGE:SEXM	-0.003	0.007	253	-0.409	0.683

LS means estimate

tRel	lsmean	SE	df	t.ratio	p.value
w-48	0.331	0.037	253	8.922	0.000
w-96	0.330	0.037	253	8.888	0.000
w-120	0.308	0.037	253	8.293	0.000
w-144	0.313	0.037	253	8.423	0.000

Effect size estimate -- omega_squared

Parameter	Omega2_partial	CI	CI_low	CI_high
tRel	0.001	0.9	0.000	0.003
AGE	-0.004	0.9	0.000	0.000
SEX	-0.004	0.9	0.000	0.000
MSONSET	-0.004	0.9	0.000	0.000
log10.RESIDVOL	0.013	0.9	0.001	0.039
ARM	0.025	0.9	0.000	0.057
AGE:SEX	-0.003	0.9	0.000	0.000

Effect size estimate -- eta_squared

Parameter	Eta2_partial	CI	CI_low	CI_high
tRel	0.002	0.9	0.000	0.006
AGE	0.000	0.9	0.000	0.000
SEX	0.000	0.9	0.000	0.012
MSONSET	0.000	0.9	0.000	0.013
log10.RESIDVOL	0.016	0.9	0.002	0.043
ARM	0.037	0.9	0.003	0.074
AGE:SEX	0.001	0.9	0.000	0.015

3) Pre-lesion vs Overall NAWM

Mixed model estimate

	Value	Std.Error	DF	t.value	p.value
(Intercept)	-0.068	0.215	1881	-0.318	0.751
tRelw-96	-0.002	0.015	1881	-0.119	0.905
tRelw-120	-0.022	0.015	1881	-1.470	0.142
tRelw-144	-0.029	0.015	1881	-1.982	0.048
AGE	0.005	0.004	253	1.276	0.203
SEXM	0.181	0.238	253	0.760	0.448
MSONSET	-0.002	0.007	253	-0.364	0.716
log10.RESIDVOL	0.247	0.076	366	3.254	0.001
ARMBIIB017 125 mcg every 2 weeks	0.139	0.111	253	1.251	0.212
ARMBIIB017 125 mcg every 4 weeks	-0.016	0.100	253	-0.164	0.870
ARMPlacebo -> BIIB017 125 mcg every 4 weeks	0.166	0.112	253	1.479	0.141
AGE:SEXM	-0.008	0.007	253	-1.177	0.240

LS means estimate

tRel	lsmean	SE	df	t.ratio	p.value
w-48	0.625	0.038	253	16.376	0.000
w-96	0.623	0.038	253	16.330	0.000
w-120	0.603	0.038	253	15.808	0.000
w-144	0.595	0.038	253	15.610	0.000

Effect size estimate -- omega_squared

Parameter	Omega2_partial	CI	CI_low	CI_high
tRel	0.001	0.9	0.000	0.004
AGE	0.000	0.9	0.000	0.013
SEX	0.001	0.9	0.000	0.018
MSONSET	-0.002	0.9	0.000	0.000
log10.RESIDVOL	0.022	0.9	0.004	0.053
ARM	0.014	0.9	0.000	0.037
AGE:SEX	0.002	0.9	0.000	0.019

Effect size estimate -- eta_squared

Parameter	Eta2_partial	CI	CI_low	CI_high
tRel	0.003	0.9	0.000	0.007
AGE	0.004	0.9	0.000	0.028
SEX	0.005	0.9	0.000	0.030
MSONSET	0.002	0.9	0.000	0.020
log10.RESIDVOL	0.024	0.9	0.005	0.057
ARM	0.025	0.9	0.000	0.055
AGE:SEX	0.005	0.9	0.000	0.031

Supplementary Material 4. Composite data for Figure 5D-F

1. Univariate analysis – Pairwise nT2 intensity differences in ASCEND SPMS trial population

1) Pre-lesion vs Contralateral NAWM

Mixed model estimate

	Value	Std.Error	DF	t.value	p.value
(Intercept)	0.201	0.031	695	6.476	0.000
tRelw-48	0.012	0.025	695	0.479	0.632
tRelw-72	0.015	0.029	695	0.535	0.593
tRelw-96	0.028	0.034	695	0.824	0.410

LS means estimate

tRel	lsmean	SE	df	t.ratio	p.value
w-24	0.201	0.031	153	6.476	0.000
w-48	0.213	0.033	153	6.369	0.000
w-72	0.217	0.036	153	5.961	0.000
w-96	0.229	0.041	153	5.656	0.000

Effect size estimate -- omega_squared

Parameter	Omega2_partial	CI	CI_low	CI_high
tRel	-0.003	0.9	0.000	0.000

Effect size estimate -- eta_squared

Parameter	Eta2_partial	CI	CI_low	CI_high
tRel	0.001	0.9	0.000	0.003

2) Contralateral versus Overall NAWM

Mixed model estimate

	Value	Std.Error	DF	t.value	p.value
(Intercept)	0.378	0.034	695	11.150	0.000
tRelw-48	-0.047	0.018	695	-2.689	0.007
tRelw-72	-0.094	0.020	695	-4.656	0.000
tRelw-96	-0.171	0.024	695	-7.232	0.000

LS means estimate

tRel	lsmean	SE	df	t.ratio	p.value
w-24	0.378	0.034	153	11.150	0.000
w-48	0.330	0.035	153	9.413	0.000
w-72	0.284	0.036	153	7.773	0.000
w-96	0.206	0.039	153	5.347	0.000

Effect size estimate -- omega_squared

Parameter	Omega2_partial	CI	CI_low	CI_high
tRel	0.072	0.9	0.041	0.102

Effect size estimate -- eta_squared

Parameter	Eta2_partial	CI	CI_low	CI_high
tRel	0.076	0.9	0.045	0.106

3) Pre-lesion vs Overall NAWM

Mixed model estimate

	Value	Std.Error	DF	t.value	p.value
(Intercept)	0.558	0.035	695	15.903	0.000
tRelw-48	-0.031	0.020	695	-1.509	0.132
tRelw-72	-0.076	0.023	695	-3.265	0.001
tRelw-96	-0.142	0.027	695	-5.168	0.000

LS means estimate

tRel	lsmean	SE	df	t.ratio	p.value
w-24	0.558	0.035	153	15.903	0.000
w-48	0.528	0.037	153	14.400	0.000
w-72	0.482	0.038	153	12.557	0.000
w-96	0.417	0.041	153	10.159	0.000

Effect size estimate -- omega_squared

Parameter	Omega2_partial	CI	CI_low	CI_high
tRel	0.037	0.9	0.015	0.059

Effect size estimate -- eta_squared

Parameter	Eta2_partial	CI	CI_low	CI_high
tRel	0.041	0.9	0.018	0.065

2. Multivariate analysis – Pairwise nT2 intensity differences in ASCEND SPMS trial population

1) Pre-lesion vs Contralateral NAWM

Mixed model estimate

	Value	Std.Error	DF	t.value	p.value
(Intercept)	0.272	0.250	695	1.086	0.278
tRelw-48	0.012	0.025	695	0.489	0.625
tRelw-72	0.016	0.029	695	0.549	0.583
tRelw-96	0.029	0.034	695	0.844	0.399
AGE	-0.002	0.004	148	-0.482	0.631
SEXM	0.267	0.342	148	0.782	0.436
MSONSET	0.004	0.006	148	0.660	0.511
log10.RESIDVOL	-0.017	0.080	314	-0.207	0.836
ARMNatalizumab 300 mg	-0.002	0.126	148	-0.016	0.987
AGE:SEXM	-0.007	0.008	148	-0.839	0.403

LS means estimate

tRel	lsmean	SE	df	t.ratio	p.value
w-24	0.191	0.063	148	3.029	0.003
w-48	0.203	0.065	148	3.108	0.002
w-72	0.207	0.067	148	3.091	0.002
w-96	0.219	0.069	148	3.162	0.002

Effect size estimate -- omega_squared

Parameter	Omega2_partial	CI	CI_low	CI_high
tRel	-0.003	0.9	0.000	0.000
AGE	-0.002	0.9	0.000	0.000
SEX	-0.006	0.9	0.000	0.000
MSONSET	-0.004	0.9	0.000	0.000
log10.RESIDVOL	-0.003	0.9	0.000	0.000
ARM	-0.006	0.9	0.000	0.000
AGE:SEX	-0.002	0.9	0.000	0.000

Effect size estimate -- eta_squared

Parameter	Eta2_partial	CI	CI_low	CI_high
tRel	0.001	0.9	0.000	0.002
AGE	0.005	0.9	0.000	0.040
SEX	0.001	0.9	0.000	0.021
MSONSET	0.002	0.9	0.000	0.032
log10.RESIDVOL	0.000	0.9	0.000	0.006
ARM	0.000	0.9	0.000	0.015
AGE:SEX	0.005	0.9	0.000	0.040

2) Contralateral versus Overall NAWM

Mixed model estimate

	Value	Std.Error	DF	t.value	p.value
(Intercept)	-0.314	0.268	695	-1.172	0.242
tRelw-48	-0.048	0.018	695	-2.722	0.007
tRelw-72	-0.094	0.020	695	-4.677	0.000
tRelw-96	-0.172	0.024	695	-7.253	0.000
AGE	0.006	0.005	148	1.235	0.219

	Value	Std.Error	DF	t.value	p.value
SEXM	0.270	0.376	148	0.718	0.474
MSONSET	-0.005	0.006	148	-0.779	0.437
log10.RESIDVOL	0.250	0.082	314	3.035	0.003
ARMNatalizumab 300 mg	0.096	0.123	148	0.784	0.434
AGE:SEXM	-0.007	0.009	148	-0.864	0.389

LS means estimate

tRel	lsmean	SE	df	t.ratio	p.value
w-24	0.415	0.062	148	6.724	0.000
w-48	0.367	0.063	148	5.831	0.000
w-72	0.320	0.064	148	5.027	0.000
w-96	0.243	0.065	148	3.734	0.000

Effect size estimate -- omega_squared

Parameter	Omega2_partial	CI	CI_low	CI_high
tRel	0.072	0.9	0.041	0.102
AGE	-0.002	0.9	0.000	0.000
SEX	-0.003	0.9	0.000	0.000
MSONSET	-0.004	0.9	0.000	0.000
log10.RESIDVOL	0.025	0.9	0.004	0.060
ARM	-0.004	0.9	0.000	0.000
AGE:SEX	-0.002	0.9	0.000	0.000

Effect size estimate -- eta_squared

Parameter	Eta2_partial	CI	CI_low	CI_high
tRel	0.076	0.9	0.045	0.106
AGE	0.005	0.9	0.000	0.041
SEX	0.004	0.9	0.000	0.037
MSONSET	0.003	0.9	0.000	0.034
log10.RESIDVOL	0.028	0.9	0.006	0.065
ARM	0.003	0.9	0.000	0.007
AGE:SEX	0.005	0.9	0.000	0.041

3) Pre-lesion vs Overall NAWM

Mixed model estimate

	Value	Std.Error	DF	t.value	p.value
(Intercept)	0.053	0.269	695	0.198	0.843
tRelw-48	-0.031	0.020	695	-1.527	0.127
tRelw-72	-0.076	0.023	695	-3.271	0.001
tRelw-96	-0.142	0.027	695	-5.170	0.000
AGE	0.003	0.005	148	0.569	0.570
SEXM	0.328	0.390	148	0.839	0.403
MSONSET	-0.002	0.006	148	-0.266	0.791
log10.RESIDVOL	0.208	0.077	314	2.697	0.007
ARMNatalizumab 300 mg	0.128	0.127	148	1.009	0.315
AGE:SEXM	-0.010	0.009	148	-1.081	0.282

LS means estimate

tRel	lsmean	SE	df	t.ratio	p.value
w-24	0.603	0.064	148	9.453	0.000
w-48	0.573	0.065	148	8.749	0.000
w-72	0.527	0.066	148	7.937	0.000

tRel	lsmean	SE	df	t.ratio	p.value
w-96	0.462	0.068	148	6.784	0.000

Effect size estimate -- omega_squared

Parameter	Omega2_partial	CI	CI_low	CI_high
tRel	0.037	0.9	0.015	0.059
AGE	-0.007	0.9	0.000	0.000
SEX	0.005	0.9	0.000	0.040
MSONSET	-0.006	0.9	0.000	0.000
log10.RESIDVOL	0.019	0.9	0.002	0.051
ARM	-0.002	0.9	0.000	0.000
AGE:SEX	0.001	0.9	0.000	0.026

Effect size estimate -- eta_squared

Parameter	Eta2_partial	CI	CI_low	CI_high
tRel	0.041	0.9	0.018	0.065
AGE	0.000	0.9	0.000	0.005
SEX	0.011	0.9	0.000	0.056
MSONSET	0.000	0.9	0.000	0.016
log10.RESIDVOL	0.022	0.9	0.003	0.056
ARM	0.005	0.9	0.000	0.040
AGE:SEX	0.008	0.9	0.000	0.048

Supplementary Table. Demographic and disease characteristics

	Analysis population prior to new T2 lesion formation		
	**SYNERGY (RMS)**	**ADVANCE (RRMS)**	**ASCEND (SPMS)**
Number of patients with eligible new T2 ROI, *n*	85	261	154
Number of new T2 lesions eligible for analysis, *n*	183	628	469
Median age, y	35	32	46
Female, %	63.5	68.6	59.7
Mean (SD) number of baseline T1 Gd^+ lesions	4.1 (7.3)	2.7 (6.5)	1.7 (6.4)
Patients with ≥1 T1 Gd^+ lesion at baseline, %	65.9	56.9	34.4
Median baseline non-enhancing T2 hyperintense lesion volume, ml	7.8	8.5	14.0
Median baseline normalized brain volume, ml	1435.6	1591.3	1434.4

Supplementary Figure. Example of the patient-level segmentation of ROIs for the analysis of pre-lesion, overall, and spatially matched contralateral NAWM prior to new T2 lesion onset detected at Week 24 (SYNERGY trial)

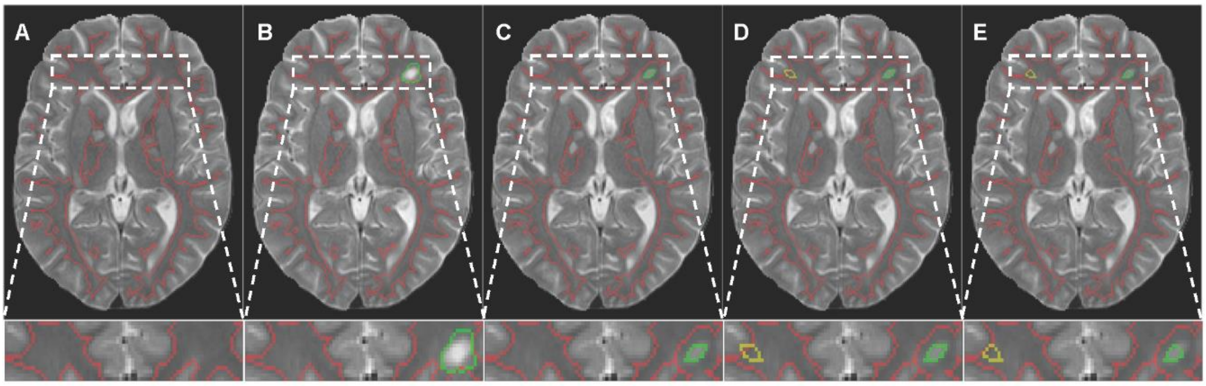

(**A**) Outline in red of NAWM as defined over the period baseline to Week 20

(**B**) The peak size in green of the new T2 lesion of interest at Week 24 (compared with Week 20)

(**C**) The portion of the new T2 lesion of interest that remains T2 hyperintense at Week 72 (residual new T2) ingreen, along with the outline in red of NAWM as defined over the period baseline to Week 72

(**D**) Contralateral ROI in yellow spatially matched to the residual new T2 lesion in green

(**E**) Modified boundaries of the contralateral spatially matched ROI in yellow such that they are completely within the longitudinal (baseline to Week 72); NAWM mask in red, with the residual new T2 boundaries in green adjusted correspondingly to match the contralateral ROI
